# Calebin A modulates inflammatory and autophagy signals for the prevention and treatment of osteoarthritis

**DOI:** 10.3389/fimmu.2024.1363947

**Published:** 2024-03-04

**Authors:** Aranka Brockmueller, Constanze Buhrmann, Parviz Shayan, Mehdi Shakibaei

**Affiliations:** ^1^Musculoskeletal Research Group and Tumor Biology, Faculty of Medicine, Institute of Anatomy, Chair of Vegetative Anatomy, Ludwig-Maximilians-University Munich, Munich, Germany; ^2^Institute of Anatomy and Cell Biology, Faculty of Medicine, University of Augsburg, Augsburg, Germany; ^3^Department of Parasitology, Faculty of Veterinary Medicine, University of Tehran, Tehran, Iran

**Keywords:** Calebin A, chondrocytes, osteoarthritis environment, inflammation, autophagy, 3D-culture

## Abstract

**Introduction:**

Osteoarthritis (OA) is associated with excessive cartilage degradation, inflammation, and decreased autophagy. Insufficient efficacy of conventional monotherapies and poor tissue regeneration due to side effects are just some of the unresolved issues. Our previous research has shown that Calebin A (CA), a component of turmeric (*Curcuma longa*), has pronounced anti-inflammatory and anti-oxidative effects by modulating various cell signaling pathways. Whether CA protects chondrocytes from degradation and apoptosis in the OA environment (EN), particularly *via* the autophagy signaling pathway, is however completely unclear.

**Methods:**

To study the anti-degradative and anti-apoptotic effects of CA in an inflamed joint, an *in vitro* model of OA-EN was created and treated with antisense oligonucleotides targeting NF-κB (ASO-NF-κB), and IκB kinase (IKK) inhibitor (BMS-345541) or the autophagy inhibitor 3-methyladenine (3-MA) and/or CA to affect chondrocyte proliferation, degradation, apoptosis, and autophagy. The mechanisms underlying the CA effects were investigated by MTT assays, immunofluorescence, transmission electron microscopy, and Western blot analysis in a 3D-OA high-density culture model.

**Results:**

In contrast to OA-EN or TNF-α-EN, a treatment with CA protects chondrocytes from stress-induced defects by inhibiting apoptosis, matrix degradation, and signaling pathways associated with inflammation (NF-κB, MMP9) or autophagy-repression (mTOR/PI3K/Akt), while promoting the expression of matrix compounds (collagen II, cartilage specific proteoglycans), transcription factor Sox9, and autophagy-associated proteins (Beclin-1, LC3). However, the preventive properties of CA in OA-EN could be partially abrogated by the autophagy inhibitor 3-MA.

**Discussion:**

The present results reveal for the first time that CA is able to ameliorate the progression of OA by modulating autophagy pathway, inhibiting inflammation and apoptosis in chondrocytes, suggesting that CA may be a novel therapeutic compound for OA.

## Graphical Abstract

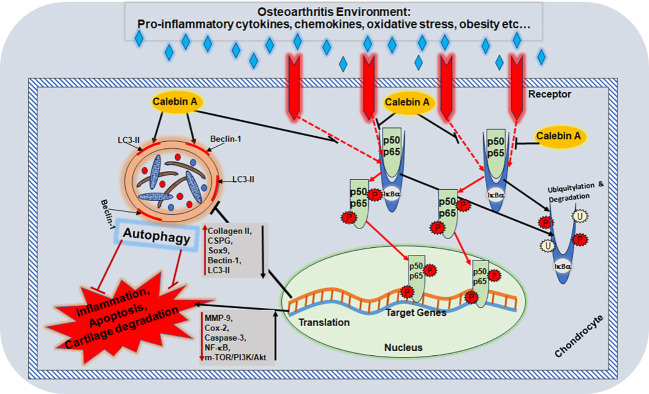

## 1 Introduction

Osteoarthritis (OA) represents the most common joint disease worldwide that affects an average of 18% of adults whereby the prevalence expands with increasing age and from 65 years onwards approximately half of the women and one-third of the men suffer from it (1). A degenerative, chronically progressive cartilage damage, accompanied by pain and functional limitations in the course, is the main issue of the disease, which may occur in any joint. Four stages are distinguished radiologically according to Kellgren–Lawrence classification from doubtful (I) to severe (IV) (2), and clinically, there are different courses such as silent, activated, or deformed. While a silent stage brings about minor problems for the patient, an activated stage results in metabolic abnormalities in articular cartilage, associated with inflammation, calcification, and destruction (3). Moreover, at the deformed stage, the articular cartilage has already disappeared in places, generating bone and tendon changes leading to joint deformities (4).

Based on the causes that trigger OA, there are primary forms by genetic predisposition as well as advanced age and secondary forms by joint dysplasia, overload, obesity, alcoholism, or metabolic disorders (5), all of which generate inflammatory events. *In vitro* and *in vivo* studies validated the cytokine, tumor necrosis factor (TNF)-α, as multifunctional key player in the pathophysiology of joint diseases such as OA, since its promotion of numerous further cytokines and enzymes activates an almost unstoppable pro-inflammatory cycle (6). Especially for the osteoarthritic synovial microenvironment, a TNF-α-forced induction of the main pro-inflammatory transcription factor, nuclear factor kappa-light-chain-enhancer of activated B-cells (NF-κB) is known (7). Persistent inflammation can quickly escalate to apoptosis in the naturally bradytrophic cartilage tissue due to its low maintenance capacity, and early defense mechanisms are necessary to stabilize cartilage homeostasis.

A crucial process here is autophagy, which breaks down dysfunctional and thus cartilage-damaging cell organelles or proteins. As part of the autophagy, joint cartilage cells activate a health-preserving and regenerative option by reducing inflammation and preventing apoptosis (8, 9). Thereby, both foreign substances and cell components to be degraded, such as discarded organelles, are taken up by autophagy and then referred to as the autophagosome. As a next step, for the purpose of dismantling, the autophagosome fuses with lysosomes and thus forms autolysosomes (10). These activities of autophagic processes are regulated by chondrocyte’s metabolism sensor, mammalian target of rapamycin’ (mTOR) (11) with linked mTOR/PI3K/Akt signaling cascade (12), and determined by a high expression of corresponding Beclin-1 and LC3-II markers in differentiated cartilage cells (9). If this protective mechanism takes effect too late or not sufficiently, apoptosis as an irreversible initiation of cell death with the consequence of OA that requires treatment results. Non-pharmacological, pharmacological, or surgical approaches are available as OA-therapeutic possibilities offering more or less satisfactory results (5). Therefore due to their lack of side effects, a supportive co-treatment with phytopharmaceuticals such as the proven grape-derived resveratrol (13, 14) or curcumin from *Curcuma longa* (13, 15) gains importance.

Calebin A (CA), another little-known but highly effective polyphenolic ingredient of *Curcuma longa*, was first isolated approximately 20 years ago (16). The cornerstone for researching its medical potential was laid by demonstrating its safe use in animal experiments, where a study on Wistar rats was carried out without any sign of toxicity despite administration of 20, 50, or 100 mg CA/kg/body weight for 3 months (17). Contrarily, an extensive health-protecting effect of this natural compound gradually crystallizes (18), and after the first demonstration of CA’s neuroprotective (16) and metabolism-modulating (19) action, a broad-based cancer-inhibiting effect was also proven. For example, the phytopharmacon suppresses growth and proliferation of gastric (20) or colorectal cancer (21, 22) cells. The inhibition of inflammatory cascades involving NF-κB and the associated interruption of disease intensification are considered to be the central mechanism of action here (22, 23).

Research into CA’s influence on the musculoskeletal system is just beginning, and at least there are first findings of CA-induced bone stabilization through downregulation of RANKL signaling suppressing osteoclastogenesis (24). Tendons close to the joint could also benefit preventively or therapeutically from a CA-associated inhibition of inflammatory NF-κB cascade as recently demonstrated by 3D *in vitro* investigations (25). To summarize, to the best of our knowledge, the effectiveness of CA has not been previously studied in relation to OA and certainly not the associated chondroprotective autophagy processes.

Considering the above, the aim of these studies was to determine whether CA could be able to modulate both inflammatory and autophagic processes in chondrocytes that are exposed to an osteoarthritis environment (OA-EN). Therefore, all experiments were performed in multicellular 3D-culture models *in vitro* simulating a lifelike inflamed joint situation.

## 2 Materials and methods 

### 2.1 Antibodies and chemical substances

As part of our experiments, the following antibodies were used: Beclin-1 (#3738) and LC3-II (#4108) from Cell Signaling Technology (Danvers, MA, USA); NF-κB (#MAB5078), MMP-9 (#MAB911), and caspase-3 (#AF835) from R&D Systems (Heidelberg, Germany); Sox9 (#TA802387) from OriGene Technologies (Herford, Germany); β-actin (#A4700), collagen type II (#AB761), and CSPG (#MAB5384-I) from Sigma-Aldrich (Taufkirchen, Germany); PI3K (#ab154598), Akt (#ab38449), and mTOR (#ab109268) from Abcam (Berlin, Germany); secondary immunofluorescence antibodies from Dianova (Hamburg, Germany); and secondary Western blot antibodies from EMD Millipore (Schwalbach, Germany). Furthermore, TNF-α was from R&D Systems (Heidelberg, Germany), 3-methyladenine (3-MA) from VWR International (Ismaning, Germany), and Epon from Plano (Marburg, Germany). BMS-345541, Fluoromount, DAPI, and MTT reagent were from Sigma-Aldrich (Taufkirchen, Germany). CA from Sabinsa Corporation (East Windsor, NJ, USA) was prepared as 5,000 µM stock in dimethyl-sulfoxide (DMSO) solution. The experimental concentrations were further diluted in cell culture medium without exceeding a DMSO concentration of 0.1%.

### 2.2 Origin and cultivation of the cells

Primary canine chondrocytes (PCHs) were isolated from cartilage samples obtained intraoperatively during joint procedures. Both the permission of the ethical committee of Ludwig-Maximilians-Universität (Munich, Germany) and the agreement of fully informed dog owner had preceded this. Fibroblasts (MRC-5) were obtained from the European Collection of Cell Cultures (Salisbury, UK), and T-lymphocytes (Jurkat) were purchased at Leibniz Institute (Braunschweig, Germany). PCH and MRC-5 grew as monolayer, while Jurkat were non-adherent cells. All cell lines were cultured in T175 cell culture flasks at 37°C and 5% CO_2_ until 70% confluency in cell culture medium containing 10% fetal bovine serum (FBS). Then, they were washed in cell culture medium containing 3% FBS (serum-starved) three times and used for experiments, all of which were done in serum-starved cell culture medium.

Dulbecco’s medium/Ham’s F-12 from Seromed (Munich, Germany) was enriched with 3% or 10% FBS and further supplemented with glutamine, penicillin/streptomycin, ascorbic acid, essential amino acids (1% each), and 0.5% amphotericin B.

### 2.3 Transient transfection

PCH were transiently transfected with antisense/sense (ASO/SO) oligonucleotides (phosphorothioate-specific) from Eurofins MWG Operon (Ebersberg, Germany). The incubation ratio was 0.5 µM ASO/SO with 10 µl/ml Lipofectin from Invitrogen (Karlsruhe, Germany). Specifically, 5′-gGAGATGCGCACTGTCCCTGGTC-3′ (ASO) corresponded to p65/NF-κB mRNA subunit, and 5′-gACCAGGGACAGTGCGCATCTC-3′ (SO) served as control substance, as described in the past (26).

### 2.4 Osteoarthritis environment

**Figure 1:**
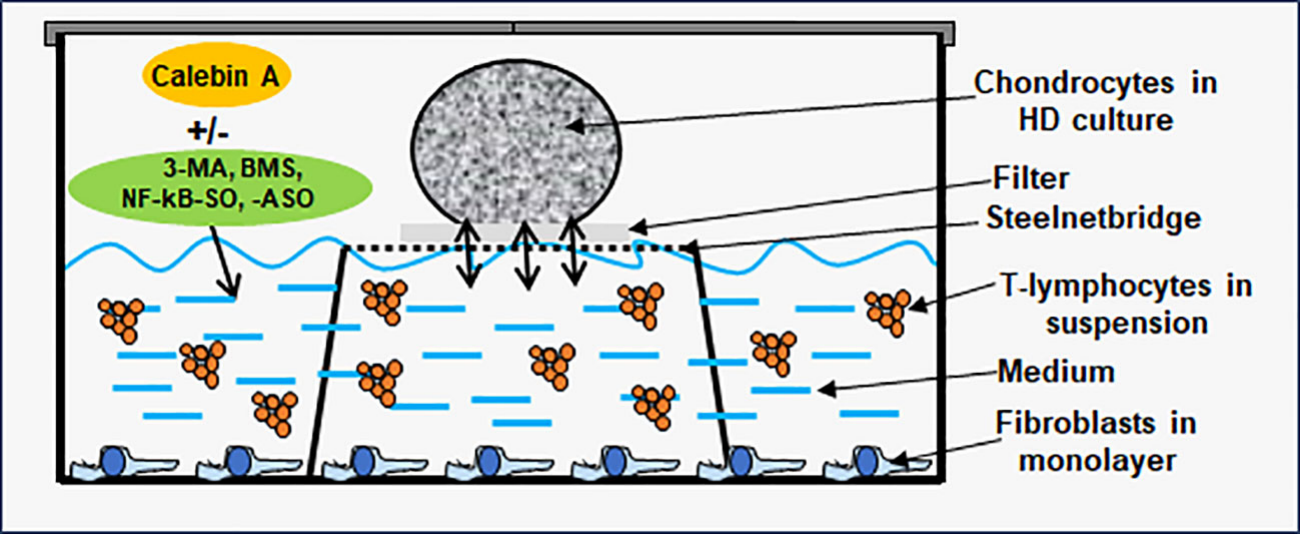
Osteoarthritis (OA) high-density (HD) environment (EN) culture model. The OA-EN was installed in well plates, where each well was equipped with a steelnet bridge and an overlying filter. Fibroblasts grew as monolayer on the bottom of the well and T-lymphocytes floated in the cell culture medium. This cell culture medium was filled up to the medium/air interface and enriched with additives such as Calebin A, 3-methyladenine (3-MA), BMS-345541 or NF-κB-SO/ASO. Chondrocytes were applied as HD culture on each filter.

To simulate an osteoarthritic joint situation *in vitro*, we constructed a multicellular OA-EN in 3D (**Figure 1**). In this context, three types of cells were combined, with PCH represented the cartilage tissue of the articular surface, MRC-5 fibroblasts represented an intact connective tissue, and Jurkat T-lymphocytes ensured the OA-associated inflammatory reaction. Therefore, PCH were cultivated as high density (HD) or coverglass culture in well plates. Additionally, fibroblasts (0.01 Mio./ml medium) were grown as monolayer on the bottom of the well plates, and Jurkat T-cells (0.01 Mio./ml medium) floated in cell culture medium suspension. The effectiveness of Jurkat cells to promote a pro-inflammatory intercellular cross-talk has been provided by a TNF-α-stimulated control (TNF-α-EN), based on previous comparisons with similar cytokines (27). The OA-EN was used in two cultivation variants for the present experiments.

#### 2.4.1 High-density culture

HD cultures were established in well plates containing a small steelnet bridge with a filter placed on top it as shown in **Figure 1** and as already used and described in detail (28, 29). The cell culture medium was filled up to the height of the filter. To start an experiment, PCH were passaged and centrifuged three times in order to obtain a liquid-free pellet. Afterwards, colonies of 2 Mio. PCH were applied to each filter (**Figure 1**) using a pipette, incubated with different treatments, and the pellets grown at the medium/air interface were evaluated. When the effect of an OA-EN was examined, a fibroblast monolayer was seeded on the bottom, and the cell culture medium was enriched with T-lymphocytes as previously described.

#### 2.4.2 Coverglass culture

For coverglass cultures, PCH (5,000 cells/coverglass) were seeded on small, round coverglasses as published in the past (15). After 24 h, the coverglasses were placed on small stellnet bridges in well plates. To initiate an OA-EN, fibroblasts were grown on the bottom and T-lymphocytes floated in suspension with cell culture medium as described before. The treatments were added for 4 h, and then, the coverglasses were fixed with methanol before they were frozen at −20°C.

### 2.5 Immunofluorescence

For immunofluorescence investigation, PCH on coverglass cultures were processed as described earlier (15). After defrosting, the PCH on coverglasses were washed with Triton solution (0.5%) and bovine serum albumin (1%) in Hank’s salt solution and incubated overnight with a primary antibody (1:80 diluted) at 4°C in a humidity chamber. One day later, the coverglasses were incubated with a secondary antibody (rhodamine-coupled, 1:100 diluted) for 2 h and stained with DAPI for 15 min to distinguish between viable and apoptotic PCH. Lastly, an embedding in Fluoromount and evaluation with a DM2000 microscope from Leica (Wetzlar, Germany) was carried out. Thereby, 400–500 PCH from 15 microscopic areas were counted and evaluated.

### 2.6 MTT assay

To compare the effect of various treatments on the viability of PCH, a MTT assay was chosen. Therefore, PCH were HD cultured, and after 3 days, the cell pellet was detached from the filter and washed in Hank’s salt solution for three times to ensure that only PCH were evaluated. Next, the PCH-pellet was dissolved in sodium citrate solution (55 mM) and centrifuged and resuspended in MTT medium (with 3% FBS, without phenol red/vitamin C). Then, as explained earlier (15, 30), 100 µl of this suspension and 10 µl of MTT solution were pipetted into each well of a 96-well plate. After 3 h, the reaction was stopped by addition of 100 µl MTT solubilization solution each, and the optical density (OD) at 550 nm was evaluated with an ELISA reader from Bio-Rad (Munich, Germany).

### 2.7 Transmission electron microscopy

Ultrastructural analysis was carried out by transmission electron microscopy (TEM) as described before (22, 30). In short, PCH were grown in HD cultures, and the cell pellet was removed from the filter after 3 days. The cells were washed for three times in Hank’s salt solution to rule out contaminations with other cell types. Thereafter, a fixation in osmium tetroxide for 2 h, an alcohol-induced dehydration, and an embedding in Epon were done. The resulting grids were cut with an Ultracut E from Reichert-Jung (Darmstadt, Germany) and uranyl-acetate/lead-citrate contrasted before evaluated by a TEM 10 microscope from Zeiss (Jena, Germany). To quantify the number of apoptotic cells, 250 cells from 20 different microscopic areas were counted.

### 2.8 Western blot

To generate Western blot samples, PCH were cultured as HD cultures, and after 7–10 days, the cell pellets were removed. Ensuring pure PCH samples, the cells were washed in Hank’s salt solution for three times. The following procedure was as previously described (15, 22). First, the cells were treated with lysis buffer and centrifuged at 4°C and 10,000 rpm for 30 min. Then, their supernatants were frozen (−80°C) overnight and prepared with an Interchim Protein Quantification Kit (Montlucon Cedex, France) and 2-mercaptoethanol the next day. SDS-PAGE Western blottings were carried out with a BIO-RAD transblot apparatus (Munich, Germany). Therefore, sample-stocked nitrocellulose membranes from Fisher Scientific (Schwerte, Germany) were incubated in blocking buffer for 2 h, then incubated in primary antibodies (1:10,000 diluted) overnight, and finally incubated in secondary antibodies (1:10,000 diluted) for 1.5 h. For densitometric evaluation, QUANTITY ONE program from BIO-RAD (Munich, Germany) was used.

### 2.9 Statistics

All experiments including their evaluations were carried out three times, and the data presented in the figures represent their average results. In this relation, all data were evaluated by Student’s t-test and *post-hoc* ANOVA with SPSS software from IBM (Ehningen, Germany). After determination of percentage effects and 95% confidence intervals, p-values <0.05 were considered as statistically significant.

## 3 Results

In this study, we designed a multicellular pro-inflammatory high-density and *vivo*-mimicking osteoarthritic environment (OA-EN) to investigate the effect of CA focused on inflammation and autophagy signaling on the suppression of OA-EN cross-talk (**Figure 1**).

### 3.1 Calebin A, similar to a specific IKK inhibitor or a specific ASO against NF-κB, suppresses the downregulation of chondrocyte viability triggered by OA-EN, but not in the presence of an autophagy-inhibitor, as shown by the MTT assay

**Figure 2:**
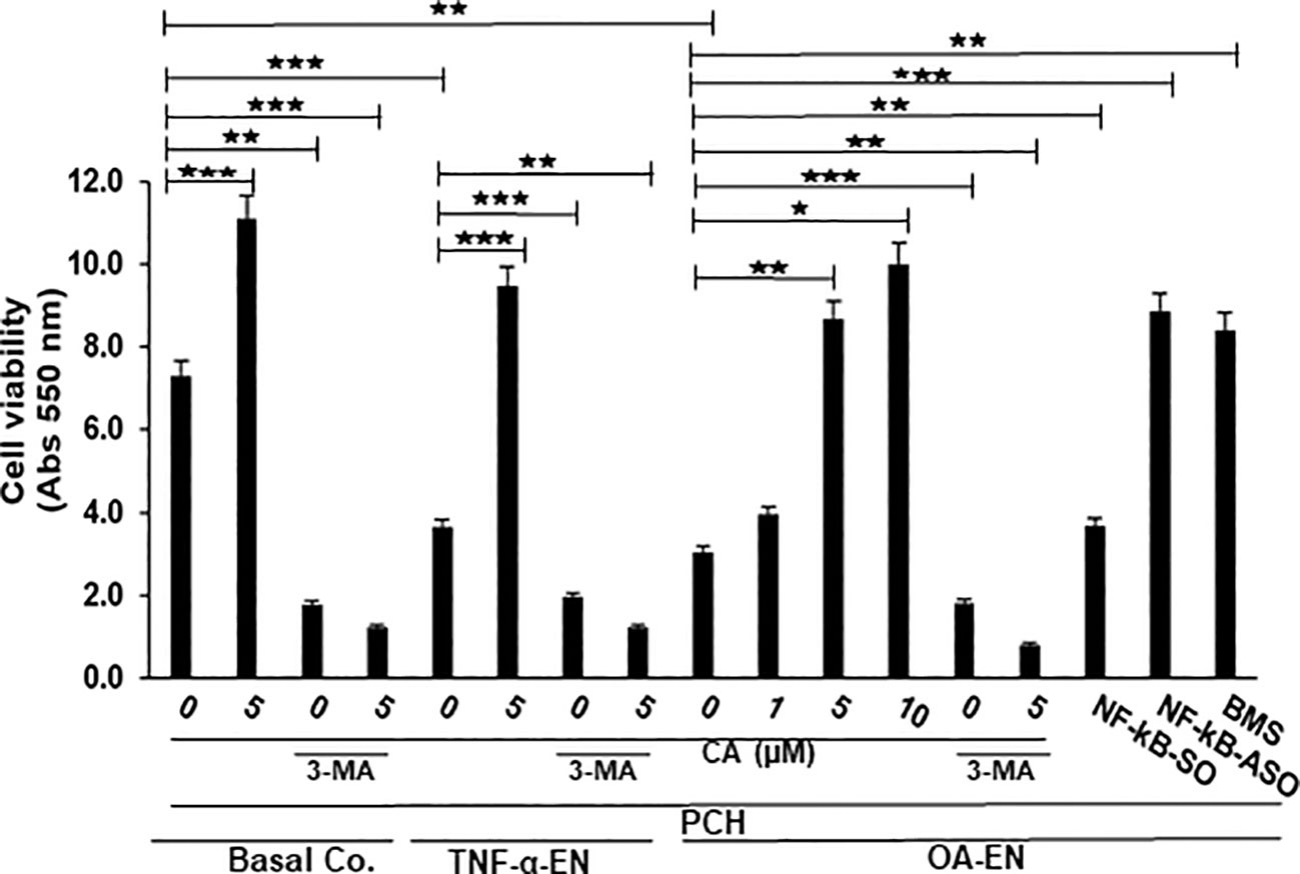
Impact of Calebin A (CA) on inflammation-triggered chondrocytes (PCH). PCH were high-density cultured as inflammation-free basal control (Basal Co.), TNF-α environment (TNF-α-EN), or osteoarthritis environment (OA-EN) and treated without additives or with supplementation of 1 µM, 5 µM, or 10 µM CA; 10 mM 3-methyladenine (3-MA); 0.5 µM NF-κB-SO/ASO; or 5 µM BMS-345541 as demonstrated on the x-axis. MTT measuring at 550 nm optical density represented PCH cell viability as shown on the y-axis. The values were given as mean ± SD, n = 3. *p < 0.05; **p < 0.01; ***p < 0.001 were classified as statistically significant.

Given the established anti-inflammatory effect of the phytopharmaceutical across diseases, PCH were cultured in 3D-HD settings as basal control, in a pro-inflammatory (TNF-α-EN or OA-EN) or in an autophagy-inhibited (3-MA) microenvironment and observed with or without the addition of CA. Furthermore, the role of inflammation in OA-EN was measured by blocking NF-κB using transient transfection (NF-κB-ASO) or specific IKK inhibitor (BMS-345541). The viability of chondrocytes exposed to these different conditions was then assessed and statistically compared using the MTT assay (**Figure 2**).

First, it became apparent that a CA treatment (5 µM) in an inflammation-free medium increased the number of viable PCH by more than half compared to the untreated, basal control, but this was abrogated after suppression of autophagy. After treatment of the basal control with 10 mM 3-MA, only 38% of viable PCH were measured. The contrast were even greater in the CA-treated basal control, where only 10% of viable chondrocytes was detectable through the addition of 3-MA (**Figure 2**).

In the next step, an inflammatory environment was created using the pro-inflammatory cytokine TNF-α. In this TNF-α-EN, 47% fewer PCH survived the treatment period than in the basal control. However, an addition of 5 µM CA to the TNF-α-EN resulted in more than a doubling of the number of viable cells. Here, too, the inhibition of autophagy had a significant effect, as the addition of 3-MA (10 mM) resulted in the survival of 51% of the PCH compared to the TNF-α control. In the CA-treated TNF-α-EN, even only 11% of the chondrocytes remained viable in the autophagy-downregulated situation (**Figure 2**). Subsequently, the OA-EN containing fibroblasts and T-lymphocytes was established for viability determination as described in *Material and methods*. Probably due to its strong inflammatory effect, 60% fewer cells remained viable in the OA-EN than in the basal control. Interestingly, a CA supplementation led to a concentration-dependent enhancement of PCH viability. While a treatment with 1 µM CA resulted in an increase of 27%, a dose of 5 µM CA led to almost a tripling of viability. At long last, an enrichment of OA-EN with 10 µM CA still slightly exceeded this (**Figure 2**), so that we determined 5 µM CA as the optimal experimental concentration.

In order to include the self-repair capacity, chondrocytes in the OA-EN were furthermore treated with 10 mM 3-MA, acting as an autophagy inhibitor. The inhibition of autophagy resulted in a significant loss of 40% PCH viability compared to OA-EN control, and a CA supplementation failed to prevent this (**Figure 2**). This became visible by a detailed examination of the CA-treated OA-EN because after the addition of 3-MA, only 9% viable PCH were found in comparison to the 3-MA-free CA-OA-EN.

Finally, the role of inflammation in OA development was illuminated. When the OA-EN was treated with the transfection control substance NF-κB-SO (0.5 µM), the PCH viability was comparable to the OA-EN control. However, when the main inflammatory transcription factor NF-κB was eliminated by transient transfection with NF-κB-ASO (0.5µM), the number of viable cells more than doubled, and this was confirmed by NF-κB blocking with the specific inhibitor BMS-345541 (**Figure 2**).

All considered, inflammatory processes and the associated downregulation of autophagy limited PCH viability, while a CA treatment was able to reverse these processes and thus promote the PCH viability. These results indicate that inhibition of inflammation and concomitant promotion of autophagy may be one of the key mechanisms of CA as anti-OA agent.

### 3.2 Calebin A promoted OA-EN-inhibited autophagic protein expression and protected chondrocytes from apoptosis, but not in the presence of an autophagy-inhibitor, as shown by immunofluorescence

**Figure 3:**
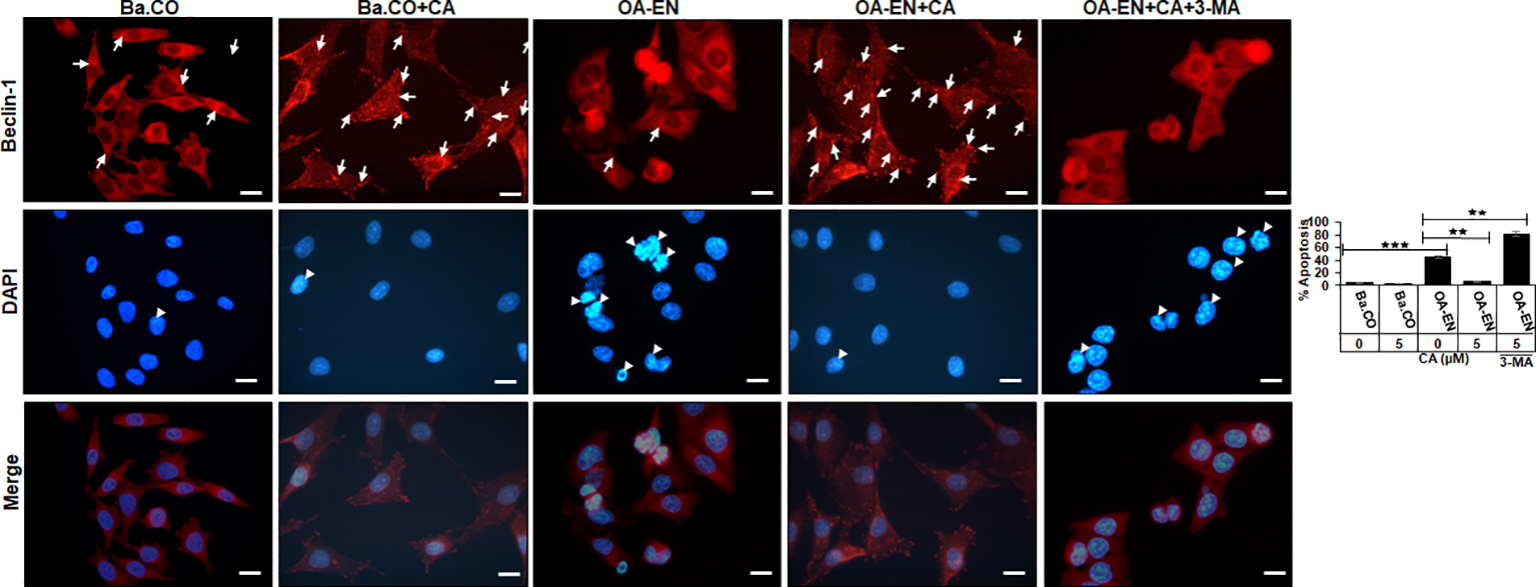
Impact of Calebin A (CA) on immunocytochemical localization of Beclin-1 in chondrocytes (PCH). PCH were incubated as basal control (Ba.CO) or osteoarthritis environment (OA-EN) as coverglass cultures and left treatment free or treated with 5 µM CA with or without 10 mM 3-methyladenine (3-MA). Immunofluorescence investigation was carried out after labeling with anti-Beclin-1 antibody (upper row, red), staining with DAPI (middle row, blue), and their merging (lower row, red and blue). A scale bar corresponds to 30 µm. The statistic chart includes x-axis (treatments) and y-axis (apoptosis rate in %). The values were given as mean ± SD, n = 3. **p < 0.01, ***p < 0.001 were classified as statistically significant.

As a bradytrophic tissue, articular cartilage relies on self-repair mechanisms. Autophagic processes are particularly important in this respect, and for this reason, the next step was to investigate whether CA influences this early alert system in OA. Therefore, PCH were grown in coverglass cultures without or with OA-EN and treated without or with CA and/or 3-MA. Afterwards, the immunocytochemical localization of autophagy marker Beclin-1 (**Figure 3**, upper row) and the viability-indicating DAPI staining (**Figure 3**, middle row) were evaluated by immunofluorescence microscopy, both individually and as a merge view (**Figure 3**, lower row).

In the inflammation-free basal control, PCH showed a moderate but even Beclin-1 labeling with strong-adhesion pseudopodia and marginally detectable apoptosis. The apoptosis rate remained low when CA was added to the basal control, but the autophagy-indicating labeling changed, as numerous strongly marked punctate autophagic vesicles were now manifested here (**Figure 3**).

An initiation of the multicellular OA-EN led to a significantly different result, as hardly any stabilizing pseudopodia were visible. In addition, 40% of apoptotic PCH were found, representing cell death, which was particularly outstanding in the merge view. Interestingly, a CA supplementation of OA-EN led to an 80% suppression of this apoptosis, the rate of which almost reached the initial value of the basal control. Moreover, numerous strongly Beclin-1 marked vesicles and a distinct pseudopodia-rich autophagic phenotype become obvious. In order to ensure that the observed CA-induced effects aimed on PCH autophagy, these self-protection processes were switched off using the autophagy-blocker 3-MA, with the result of a very high apoptosis rate (80%) and a non-significant effect of CA treatment (**Figure 3**).

Overall, these results show that inflammatory processes in PCH limit autophagy activity, leading to a predominance of apoptosis. However, CA treatment reduces inflammation, promotes autophagy, and suppresses apoptosis, resulting in a PCH-promoting equilibrium under OA conditions.

### 3.3 Calebin A abolishes OA-EN-induced apoptosis, degradation of chondrocytes and promotes autophagosomes, as shown by transmission electron microscopy

**Figure 4:**
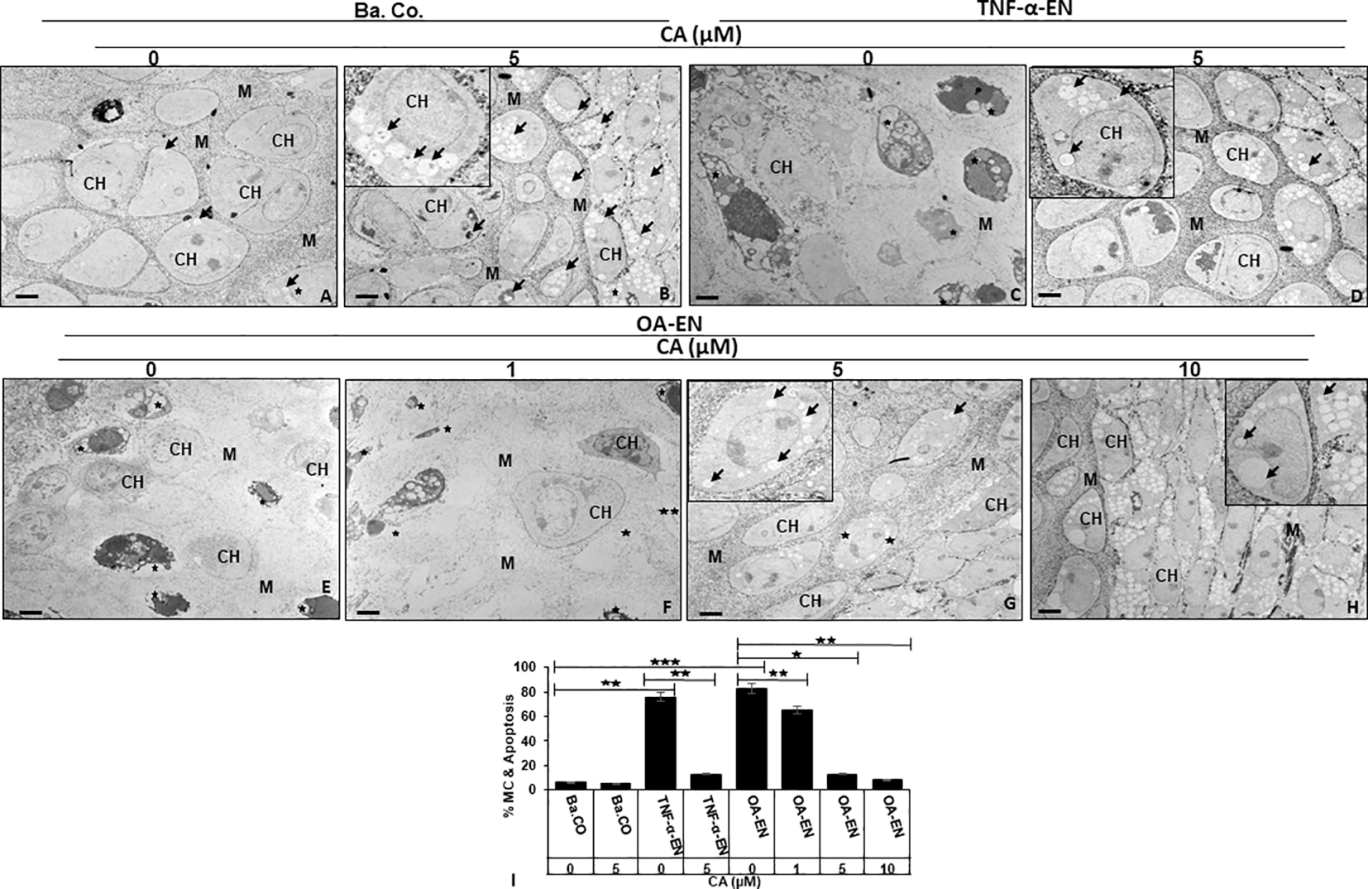
Impact of Calebin A (CA) on chondrocyte (PCH) ultrastructure focusing autophagy-apoptosis interplay. PCH were high density (HD) cultured without inflammation (basal control, Ba.Co., **A, B**), in TNF-α environment (TNF-α-EN, **C, D**), or in osteoarthritis environment (OA-EN, **E–H**). The cultures were left treatment-free or treated with 1, 5, or 10µM CA and thereafter investigated by transmission electron microscope (TEM). Marking: CH, chondrocyte; M, matrix; arrow, autophagic vesicle/autophagosome/autolysosome; star, mitochondrial changes/apoptosis. Magnification: **(A–H)** ×5,000; scale bar = 1 μM. Insets: ×15,000. The statistic diagram **(I)** shows different treatments (x-axis) and the rate of mitochondrial changes (MC) and apoptosis in % (y-axis). The values were given as mean ± SD (n = 3). *p < 0.05, **p < 0.01, ***p < 0.001 were classified as statistically significant.

To assess the suspected interplay of autophagy and apoptosis in detail at the ultrastructural level, HD-PCH cultures were maintained without (basal control) or with (TNF-α-EN/OA-EN) inflammatory conditions and with/without the addition of CA. The evaluation of chondrocyte’s ultrastructure was then carried out by TEM (**Figure 4**).

The observation of the basal control revealed intact PCH with a smooth surface embedded in a well-organized extracellular matrix (ECM), few autophagic vesicles, and rare apoptosis (**Figure 4A**). With a constant low cell death rate, an addition of 5 µM CA to the basal control led to a marked formation of stable, pseudopodia-rich PCH containing numerous autophagosomes and autolysosomes (**Figure 4B**). In contrast, the PCH in TNF-α-EN were deformed due to inflammation and had an extraordinarily high number of 78% of cells with mitochondrial changes or even apoptotic bodies (**Figure 4C**). Interestingly, the PCH morphology significantly changed through a 5 µM CA supplementation to the TNF-α-EN, as apoptosis rate was drastically reduced to 13%, and the cells resumed their vesicle and pseudopodia-rich shape with a marked augmentation in the number of autophagosomes and autolysosomes (**Figure 4D**).

A similar sight of destroyed PCH as in treatment-free TNF-α-EN was also to be seen in treatment-free OA-EN. Here, the cells lost their stable form, and more than 80% of them were mitochondrially changed or apoptotic (**Figure 4E**). An addition of CA to the OA-EN affected concentration dependence, as while the apoptosis rate persisted at 65% during treatment of 1 µM CA, it was reduced considerably to 14% by 5 µM CA. Now, the morphology corresponded to the basal control treated with 5 µM CA and the TNF-α-EN treated with the same concentration (**Figures 4F-H**). Therefore, and because a higher dosage of 10 µM CA clearly resulted in higher autophagosome and autolysosome formation than the basal control, 5 µM CA was confirmed as the optimal concentration for our PCH studies. Altogether, the inflammatory environment prevented autophagy and simultaneously induced apoptosis in chondrocytes and a degradation of the ECM. The natural polyphenol CA was able to modulate the interplay of autophagy and apoptosis and stabilized chondrocytes and ECM synthesis by at least promoting autophagy and suppressing inflammation, mitochondrial changes, and apoptosis.

### 3.4 Calebin A maintains the functionality of chondrocytes and protects them from pathological processes in OA-EN, but not in the presence of an autophagy inhibitor

After noticing large possibilities of CA for autophagy modulation, we wanted to clarify these detailed effects on molecular processes in chondrocytes. Therefore, PCH were HD cultivated as basal control or in OA-EN, where OA-EN was carried out as control or treated with 5 µM CA, 10 mM 3-MA, or a combination of both and the subsequent evaluation occurred by Western blotting.

**Figure 5:**
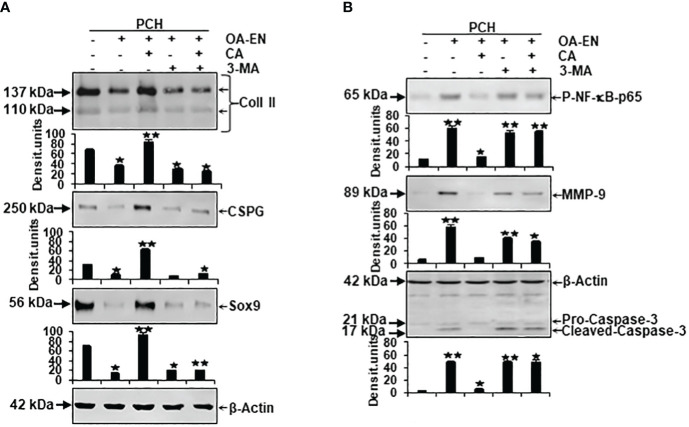
Impact of Calebin A (CA) on the physiological and pathological protein expression in chondrocytes (PCH). x-axis: PCH from high density (HD) cultures grew as basal control or in osteoarthritis environment (OA-EN) without treatment or with an addition of 5 µM CA, 10 mM 3-MA, or both combined. y-axis: Western blotting was evaluated taking densitometric units into account with, related to OA-EN control, *p<0.05 and **p<0.01 (n = 3). **(A)** The protein expression of extracellular matrix (ECM; collagen type II, CSPG) and chondrocyte-specific transcription factor (Sox9) was investigated. **(B)** Then, the levels of inflammation (p-NF-κB-p65), cell degradation (MMP-9), and apoptosis (cleaved-caspase-3) were examined. (A+B) β-actin was used as housekeeping control.

First, an indication of the PCH viability was provided by examination of their essential ECM components collagen type II (Coll II) and chondroitin sulfate proteoglycan (CSPG). Compared to basal control, both parameters decreased due to the cultivation of the chondrocytes in OA-EN. However, an addition of CA led to a significant increase in Coll II and CSPG, so that their expression was not only higher than in treatment-free OA-EN but also significantly stronger than in the basal control. Interestingly, an inhibition of autophagic pathways by 3-MA ensured low levels of Coll II and CSPG, and this was not reversible by CA supplementation (**Figure 5A**). Furthermore, the chondrogenic transcription factor Sox9 was very impressively influenced in the same way because its solid expression found in the basal control was massively downregulated by OA-EN-induced inflammation. A treatment of PCH with CA interrupted this tendency and upregulated Sox9 at a value exceeding that of the basal control. Also for this parameter, a suppression of autophagy *via* 3-MA addition leads to a decline in expression, which could not be reversed by CA (**Figure 5A**).

Next, inflammation marker p-NF-κB-p65, cell-degradation-associated MMP-9, and apoptosis indicator cleaved-caspase-3, representing possible pathological signaling processes in chondrocytes, were considered in a differentiated manner. As expected, the expression of phosphorylated and thereby activated inflammatory p-NF-κB-p65 was significantly expanded by OA-EN in contrast to the basal control. A CA treatment of OA-EN suppressed this inflammation markedly, confirming CA’s extensive anti-inflammatory impact, whereas the addition of autophagy-hampering 3-MA entailed an increase in p-NF-κB-p65 that was not annullable by CA (**Figure 5B**). In line with this, the inflammation-accompanying degradation and degradation-resulting apoptosis also reproduced this dynamic tendency. Both parameters were significantly forced in OA-EN and downregulated due to CA supplementation, but not in the presence of 3-MA (**Figure 5B**).

All in all, these findings summarize a physiology-restricting and pathology-promoting influence of the osteoarthritic environment but confirm CA’s PCH-supporting possibilities, which are noticeable, among other things, through the modulation of ECM components, integral transcription factors, inflammatory cascades, and the induction of cell degradation and apoptosis.

### 3.5 Calebin A disrupts the impaired expression of autophagy-specific markers and autophagy-inhibitory signaling pathways regulated by OA-EN in chondrocytes, but not in the presence of an autophagy inhibitor

**Figure 6:**
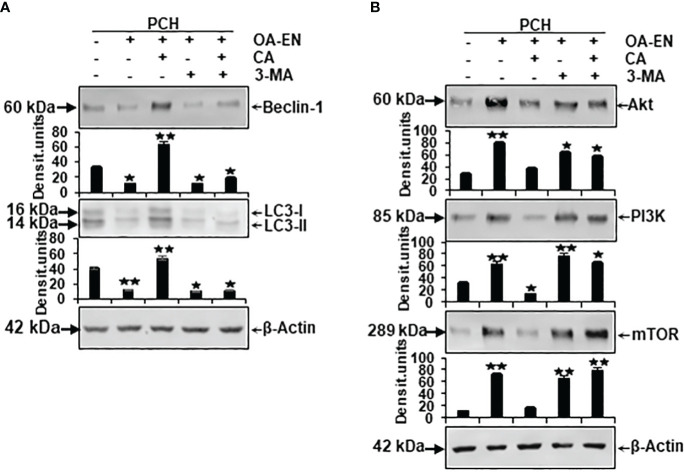
Impact of Calebin A (CA) on the expression of autophagy-specific markers and autophagy-associated signaling pathways in chondrocytes (PCH). x-axis: high-density (HD) cultured PCH were treated as basal control or in osteoarthritis environment (OA-EN) without supplementation or with of 5 µM CA, 10 mM 3-MA, or a combination thereof. y-axis: densitometric units of Western blots were evaluated with *p<0.05 and **p<0.01, (n = 3) compared to OA-EN control. The protein expression of autophagy-specific parameters (**A**, Beclin-1, LC3-II) and signaling pathways (**B**, Akt, PI3K, mTOR) was demonstrated, and β-actin **(A, B)** served as housekeeping control.

To finally confirm the results described, the Western blot evaluation was profoundly investigated. For this reason, the samples explained in the previous subchapter were immunoblotted with antibodies against autophagy-related marker proteins and signaling molecules associated with autophagy pathways in chondrocytes (**Figure 6**).

Concerning this matter, the known autophagic benchmarks Beclin-1 and LC3-II were found to be clearly expressed in PCH from basal control and comparatively downregulated in the OA-EN. Then, very interesting and never shown before, a CA supplementation of PCH lead to a significant upregulation of both autophagy parameters despite OA-EN. In the following, PCH treated with the autophagy-inhibitor 3-MA showed a marginal expression of both, Beclin-1 and LC3-II, regardless of whether CA has been added to OA-EN or not. These findings confirmed the specificity of 3-MA as an anti-autophagy protein on the one hand and demonstrated the dependence of CA’s effect on intact autophagy mechanisms in PCH on the other hand (**Figure 6A**).

At long last, the investigation of mTOR, PI3K, and Akt proteins completed the experiments, as their axis is known to be induced by inflammatory processes and to prevent autophagy in chondrocytes as a consequence thereof. All three proteins were found in small quantities in the basal control but, as expected, highly concentrated in PCH after OA-EN cultivation. This overexpression of mTOR, PI3K, and Akt could be mitigated by CA addition to the inflamed chondrocytes. However, if the PCH were treated with 3-MA, the chondrocytes lacked a physiological counterpart and the inflammatory processes dominated, which was reflected in an increased level of the mTOR/PI3K/Akt cascade and could not be reversed by CA (**Figure 6B**).

In summary, these Western blot evaluations confirm the inflammation promotion and autophagy suppression of OA-EN but underline the importance of autophagy signaling pathways in CA’s physiology-regulating effects in chondrocytes.

## 4 Discussion

OA is a widespread disease in society as a whole (1), which leads to progressive, irreversible, and only symptomatically treatable limitations in the everyday lives of affected people (5). It would therefore be important from both an economic and a patient perspective to reduce or at least limit the OA-induced joint damages. Against this background of a great need for the further development of preventive or therapeutic strategies, complementary treatment options involving natural phytopharmaceuticals such as grape-ingredient resveratrol (13, 14) or turmeric-derived curcumin (15, 31) are discussed. As CA, a fairly novel turmeric component, shows more and more a wide-ranging potential against divers health issues (18, 23), we wanted to examine its suitability in this respect. Therefore, multicellular culture models were established, and CA’s influences on OA-EN (**Figure 1**) stressed chondrocytes were observed with a special focus on autophagic self-repair processes. On this occasion, the present investigations revealed following key facts: OA-EN (I) promotes inflammation, degradation, and apoptosis in PCH and (II) reduces viability and autophagy in PCH. However, CA (III) represses inflammation, pathological tendencies, and apoptosis in PCH and, in parallel, (IV) strengthens chondrocyte’s ECM, viability, physiological processes, (V) enhances their autophagic self-repair capacities, and (VI) exerts its chondroprotective abilities largely *via* regulation of autophagy.

After the implementation of OA-EN, we found that this multicellular setting had comparable effects to the TNF-α-EN on cell viability (**Figure 2**) and ultrastructure (**Figure 4**) and that an addition of CA had very similar effects in both environments. This was based on the background knowledge that the cytokine TNF-α has been known for many years as a pro-inflammatory product of T-lymphocytes and monocytes (32). As a result, it has a significant share in spreading harmful inflammation in various tissues including cartilage, where TNF-α destroys the organization of chondrocyte’s matrix (15, 33), and serves as a relevant target for natural substances. In sum, these observations pointed out the inflammatory characteristics of OA-EN confirming earlier *in vitro* results from analogical co-culture models (27, 34) and the clinical situation of inflamed OA joints (35). As a result of the chronic inflammation, OA patients develop dysfunctional, degraded, and thus apoptotic chondrocytes (36), which, interestingly, we were able to simulate with our OA-EN culture (**Figures 4**, **5**). Moreover, beyond this clinically known scenario, the present findings demonstrated a clear inflammation-associated limitation of autophagic self-repair processes in chondrocytes (**Figures 3**, **6**). Altogether, joint inflammations result in massively destructive properties that needs to be interrupted.

Based on the observed pathological effects of OA-EN on PCH, the next step was to specifically investigate the influence of the natural polyphenol CA on these cells. In this context, chondrocytes grown without an inflammatory environment (basal control) were left untreated or supplemented with CA, and a comparison with each other showed not only a non-toxicity of CA toward chondrocytes but, on the contrary, a viability promotion (**Figure 2**), autophagy support (**Figures 3**, **4**), and even apoptosis repression (**Figures 3**, **4**) turned out. These properties are obvious similar to a known chondrocyte stabilization by the structurally related turmeric component curcumin of which, by long-term research, a regulation of inflammatory, autophagic, and apoptotic signaling pathways were identified (15, 37). Interestingly, CA maintained its valuable anti-degenerative (MMP-9) and anti-apoptotic (caspase-3) features despite an initiation of OA-EN resulting in a significant anti-inflammatory (NF-κB) capacity (**Figures 4**, **5B**). A comparative and confirmatory prevention of inflammation of the phytotherapeutic in other constellations, for example, in the tumor microenvironment of cancer cells (22, 23, 38) or tumor-associated bone loss (24), underlines its modulatory potential to restore cell homeostasis and promote the natural physiology of healthy tissue.

A further important balancing mechanism for cartilage tissue is the perpetuation of an intact ECM, which consists of collagen, elastins, and proteoglycans (39), and, due to its high water binding, has both a protective and nutritional function (40). As an advantage, our results pointed to CA’s support of Coll II, CSPG, and thus ECM synthesis and the expression of cartilage-specific transcription factor Sox9 (**Figure 5A**), suggesting a holistic, broad-based positive influence on PCH despite an OA-promoting environment.

When considering CA’s modulation of chondrocyte signaling, we noticed the regulation of autophagy as a crucial interface between physiology and pathology. Autophagy represents an evolutionarily conserved intracellular homeostatic mechanism, ensuring the survival of cells such as chondrocytes by initiating the disposal of damaged cell organelles, microorganisms, and from which new energy is derived (41). These self-repair processes are particularly important against the development or spread of OA-induced joint alterations (42). The modulation of mTOR/Akt/PI3K pathway thereby acts as a key regulator related to autophagic processes in chondrocytes, and in recent studies, phytochemicals demonstrated their potential for influencing this signaling. Curcumin, for example, is able to avert cytokine-stimulated cartilage degradation by inhibiting mTOR/Akt/PI3K signals and accompanying promotion of collagen production and suppression of MMP expression (43). Moreover, isorhynchophylline, used in traditional Chinese medicine, impedes osteoarticular and chondrogenic inflammation by the upregulation of autophagic Beclin-1 and LC3-II proteins and a simultaneous downregulation of the mTOR/Akt/PI3K signaling pathway (44). Consistent with these findings, our results showed a clear CA-related promotion of autophagosomes, autolysosomes (**Figure 4**), and autophagy-specific molecular markers (Beclin-1, LC3-II), and an undoubted suppression of mTOR/Akt/PI3K pathway (**Figure 6**) in PCH with an assumed increase in their self-repair and thus OA-defense capacity. Moreover, a much more far-reaching fact was revealed: all the cartilage-promoting effects described so far could only be observed to a very weak extent in the presence of the autophagy-inhibitor 3-MA (**Figures 2**, **3**, **5**), which seems to make the modulation of autophagic processes a central mechanism of CA’s holistic chondroprotection. These insights gained into the influence of CA on chondrocytes are completely new, as to the best of our knowledge, this has never been studied before. It remains undisputed that this research work offers a very first basis, which would have to be deepened by further detailed projects. In order to be able to assess the transferability of the *in vitro* results to processes in human joints, the tolerability and bioavailability of CA must first be clarified. In this respect, a non-toxicity observed even at high doses in experiments with rats (17) appears to be motivating. The bioavailability of curcumin could serve as an indication here, which, for many years, was considered to be low, but can now be significantly increased through special purification and new formulations that lead to improved intestinal absorption (45). Overall, the credibility of our new results was strengthened not only by repeated experiments but also by other phytotherapeutics with noticeable anti-OA properties, for example, extracted from *Luzula sylvatica* (46), *Olea europaea* (40), *Uncaria rhynchophylla* (44), *Boswellia serrata*, *Curcuma longa*, or *Tamarindus indica* (47). Therefore, an establishment of plant-based supplements for OA prevention or co-therapy could represent an approach worth pursuing. Finally, it cannot be ruled out that the phytotherapeutic agent CA could close a treatment gap, since the multicellular cross-talk, which has been successfully modulated in the present results, is currently considered to have hidden therapeutic potential (48), especially with regard to the regenerative and OA-averting ability of cartilage.

## 5 Conclusion

Contemplating an increasing human life expectancy and growing OA prevalences, the need for cost-effective, abundant, and well-tolerated therapeutic options remains undisputed. Within the present study, the natural polyphenol CA demonstrated for the first time a significant upregulation of autophagosomes, autolysosomes, Beclin-1, LC3-II, Coll II, CSPG, and Sox9, and simultaneous downregulation of NF-κB, MMP-9, caspase-3, and mTOR/PI3K/Akt pathway in chondrocytes. Thereby, the control of autophagic signals crystallized as one of the key mechanisms in its multitarging, anti-inflammatory, anti-degrading, and apoptosis-preventing effects in PCH. All summarized, CA seems to be a suitable co-therapeutic agent for the prevention or treatment of OA and more in-depth research in this area, especially on a clinical level, in the future would be welcome.

## Data availability statement

The raw data supporting the conclusions of this article will be made available by the authors, without undue reservation.

## Author contributions

AB: Investigation, Methodology, Validation, Visualization, Writing – original draft, Writing – review & editing. CB: Investigation, Methodology, Writing – review & editing. PS: Writing – review & editing. MS: Conceptualization, Data curation, Supervision, Visualization, Writing – original draft, Writing – review & editing.
